# Reply to “Reconsidering dopaminergic modulation in Alzheimer's disease: A case for levodopa/carbidopa as a disease‐modifying agent”

**DOI:** 10.1002/alz.70533

**Published:** 2025-07-22

**Authors:** Zsuzsa Sárkány, Joana Damásio, Sandra Macedo‐Ribeiro, Pedro M. Martins

**Affiliations:** ^1^ i3S – Instituto de Investigação e Inovação em Saúde Universidade do Porto Porto Portugal; ^2^ IBMC – Instituto de Biologia Molecular e Celular Universidade do Porto Porto Portugal; ^3^ Centro de Genética Preditiva e Preventiva (CGPP) IBMC Universidade do Porto Porto Portugal; ^4^ Neurology Department Centro Hospitalar Universitário de Santo António, ULS de Santo António Porto Portugal; ^5^ ICBAS School of Medicine and Biomedical Sciences Universidade do Porto Porto Portugal

1

We thank Nobili and D'Amelio for endorsing our study on clinical and biomarker outcomes by participants in the National Alzheimer's Coordinating Center Uniform Data Set exposed to levodopa/carbidopa.^1^ After clearly summarizing our main conclusions and methodology, their letter further discusses the possible mechanisms by which the use of levodopa (or L‐dopa) may decrease the cerebrospinal levels of amyloid beta (Aβ)_42_ peptide, total tau, and phosphorylated tau, and delay progression to dementia. They highlight the critical role of early degeneration of the dopaminergic midbrain system in driving cognitive and non‐cognitive symptoms in Alzheimer's disease (AD) mouse models. Additional mechanisms, including the L‐dopa–mediated upregulation of neprilysin expression and the inhibition of glycogen synthase kinase‐3β, are noted to explain the observed biomarker profiles. Nobili and D'Amelio conclude that a biomarker‐enriched, randomized clinical trial (RCT) of low L‐dopa doses in prodromal AD is both warranted and timely for efficacy and tolerability assessments without the inevitable limitations of retrospective studies.

We take the opportunity to echo this letter's call for action and underscore two primary challenges posed by Phase 2 clinical trials of low‐dose L‐dopa in AD, namely the lack of funds for late‐stage clinical trials and the adverse events (AEs) associated with L‐dopa formulations originally developed for Parkinson's disease (PD). L‐dopa, combined with aromatic L‐amino acid decarboxylase (AADC) inhibitors (such as carbidopa) or catechol‐O‐methyltransferase inhibitors, is a generic drug with expired patent protection of the original formulations. This fact discourages investment by pharmaceutical companies, as the elevated costs for advanced RCTs may not yield adequate returns.^2^ As we have previously discussed,^1^ it is well established that motor complications can develop as early as 0.5 to 2 years after the initiation of chronic L‐dopa therapy in patients with PD. In fact, a recent meta‐analysis of RCTs comparing L‐dopa to other anti‐PD drugs showed that the risk of dyskinesia was significantly higher for L‐dopa and increased in a dose‐dependent manner.^3^ However, an open‐label RCT with a 7 year follow‐up found no increase in mortality associated with L‐dopa use.^4^ Our analysis of death rates aligns with this finding for the “mild cognitive impairment” and “dementia” subgroups, but not for “normal cognition.”^1^ This discrepancy was likely due to the high heterogeneity within the latter group.^5^

A potential approach to addressing the challenges of profitability, safety, and efficacy − briefly outlined in our paper − involves developing new drug formulations tailored to the milder dopamine deficiency observed in AD and spinocerebellar ataxia type 3 (SCA3, or Machado–Joseph disease). These formulations should aim to (1) deliver L‐dopa at doses lower than the adopted during early‐stage PD (typically 300 mg/day L‐dopa) and (2) include carbidopa at the minimum effective dose to inhibit peripheral AADC in adults (≈ 75 mg/day carbidopa).^1^ In vitro studies suggest that restoring physiological dopamine levels in the brains of AD and SCA3 patients is enough to inhibit the proliferative oligomerization of Aβ_42_ in AD, and ataxin‐3 in SCA3 (patent submitted).^6^ Reformulation of L‐dopa to unprecedented doses and L‐dopa:carbidopa ratios will have the 2‐fold interest of minimizing AEs and of obtaining market exclusivity for sponsors of RCTs testing the new indication. Because SCA3 is a rare disease, an orphan drug designation may additionally be assigned to the new L‐dopa formulation, thus streamlining regulatory pathways to market. In conclusion, the compelling safety and efficacy evidence alongside the urgent unmet need in AD, position low‐dose L‐dopa as a transformative opportunity for governmental agencies, philanthropies, and pharmaceutical companies to invest in Phase 2/3 RCTs to validate efficacy and optimize drug formulations.

## CONFLICT OF INTEREST STATEMENT

The authors declare the following competing interests: Z.S., S.M.R., and P.M.M. are co‐inventors in provisional patent applications for the use of low‐dose levodopa formulations for the treatment of neurodegenerative disorders. J.D. served as a consultant for Biogen and Biohaven. Author disclosures are available in the supporting information.

## Supporting information



Supporting Information
